# Endoluminal rescue of false lumen graft deployment in TEVAR for type B aortic dissection: a case report and literature review

**DOI:** 10.3389/fcvm.2024.1461511

**Published:** 2024-09-09

**Authors:** Hong Jiang Zhu, Feng Yan, Peng Peng Zhao

**Affiliations:** Department of Vascular Interventional Surgery, Zhangjiajie People’s Hospital, Zhangjiajie, Hunan, China

**Keywords:** aortic, aortic dissection, TEVAR, stent graft misdeployment, case report

## Abstract

**Background:**

Thoracic endovascular aortic repair (TEVAR) has increasingly become the preferred surgical intervention for Stanford type B aortic dissection (TBAD). The primary objective of this procedure is to seal the primary entry tear to promote positive aortic remodeling. However, the increased use of TEVAR has also led to a rise in surgical complications. Among these, the accidental deployment of the stent into the false lumen is a rare but serious complication that can result in aortic false lumen rupture and inadequate perfusion of abdominal organs.

**Case summary:**

This case report described a 78-year-old man who presented to our hospital with sudden onset chest and back pain and was subsequently diagnosed with TBAD via aortic CTA. As conventional medical therapy failed to alleviate his chest pain, the patient underwent TEVAR. During the procedure, a complication arose when the distal end of the endograft was mistakenly deployed into the false lumen, leading to insufficient perfusion of the abdominal organs. Recognizing this issue intraoperatively, an additional endograft was promptly inserted at the distal end to reroute blood flow back to the true lumen of the aorta, thereby restoring visceral perfusion. Post-intervention, the patient's chest pain improved, and he was successfully discharged from the hospital.

**Conclusion:**

Accidental deployment of a endograft into the false lumen during TEVAR is a rare but serious complication. Intraoperative angiography plays a crucial role in rapidly and accurately identifying this issue by detecting insufficient perfusion of abdominal organs. The use of intravascular ultrasound may help reduce the incidence of this complication. Endovascular repair is an effective emergency strategy to quickly redirect blood flow back to the true lumen, making it the preferred method for managing such emergencies.

## 1 Introduction

Thoracic endovascular aortic repair (TEVAR) has become the leading surgical intervention for Stanford type B aortic dissection (TBAD). This procedure involves inserting a covered stent into the true lumen to seal the primary entry tear, thereby enhancing blood flow in the true lumen and reducing pressure in the false lumen, which supports favorable aortic remodeling (1). However, the increased utilization of TEVAR has also led to a rise in surgical complications. Among these, the accidental deployment of the endograft into the false lumen is a particularly rare but serious complication. Such misplacement can result in inadequate perfusion of abdominal organs or even rupture of the aortic false lumen. In this report, we present the case of a 78-year-old man who suddenly developed chest and back pain and was ultimately diagnosed with TBAD through aortic CTA. During the TEVAR procedure, a complication arose when the distal end of the endograft was mistakenly deployed into the false lumen. Recognizing this issue intraoperatively, an additional stent was promptly placed at the distal end to redirect blood flow to the true lumen of the aorta. This is an exceedingly rare surgical complication that is seldom documented in the literature.

## 2 Case presentation

**Figure 1:**
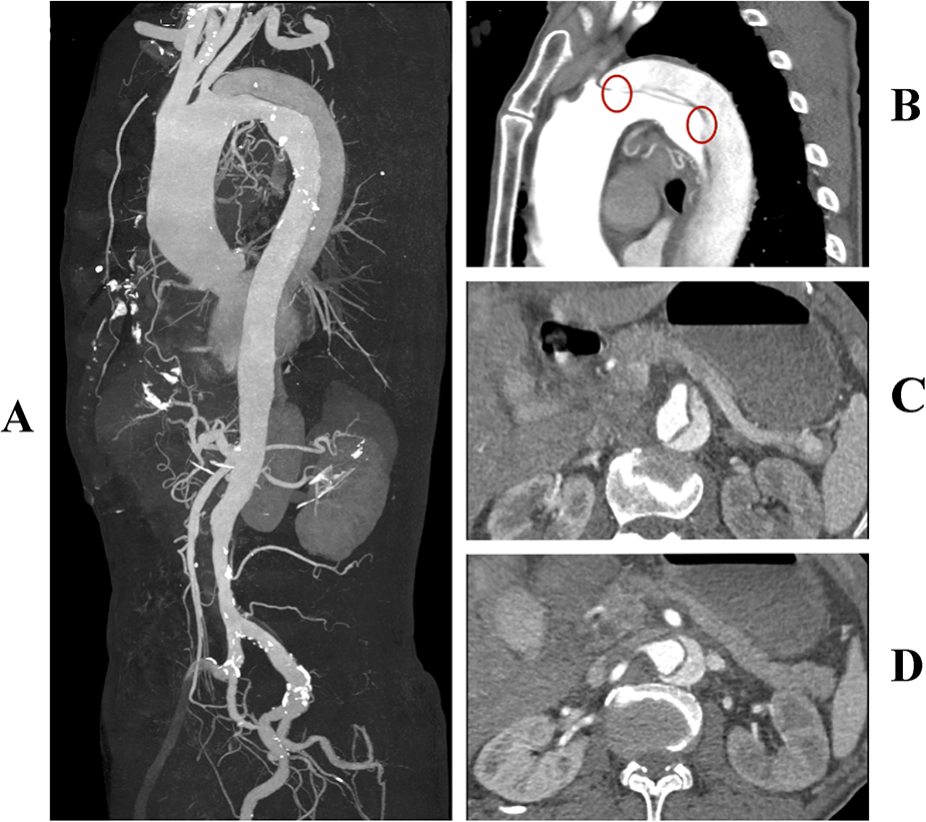
**(A)** Preoperative three-dimensional reconstruction of the aorta. **(B)** Multiple entry tears at the aortic arch, with the first tear located 8 mm distal to the origin of the left subclavian artery (highlighted by a red circle). **(C)** The superior mesenteric artery is supplied by both the true and false lumens simultaneously. **(D)** The left renal artery is supplied by the false lumen, while the right renal artery is supplied by the true lumen.

A 78-year-old man presented to our hospital with sudden onset chest and back pain. A CTA examination revealed a TBAD with multiple entry tears at zone 3 (Figures 1A,B). Both the true and false lumens were supplying blood to the superior mesenteric artery (Figure 1C). The left renal artery was supplied by the false lumen, while the right renal artery was supplied by the true lumen (Figure 1D). The patient had a history of hypertension and was regularly taking telmisartan for blood pressure control, although specific measurements were not recorded. Due to persistent chest pain, TEVAR was indicated.

**Figure 2:**
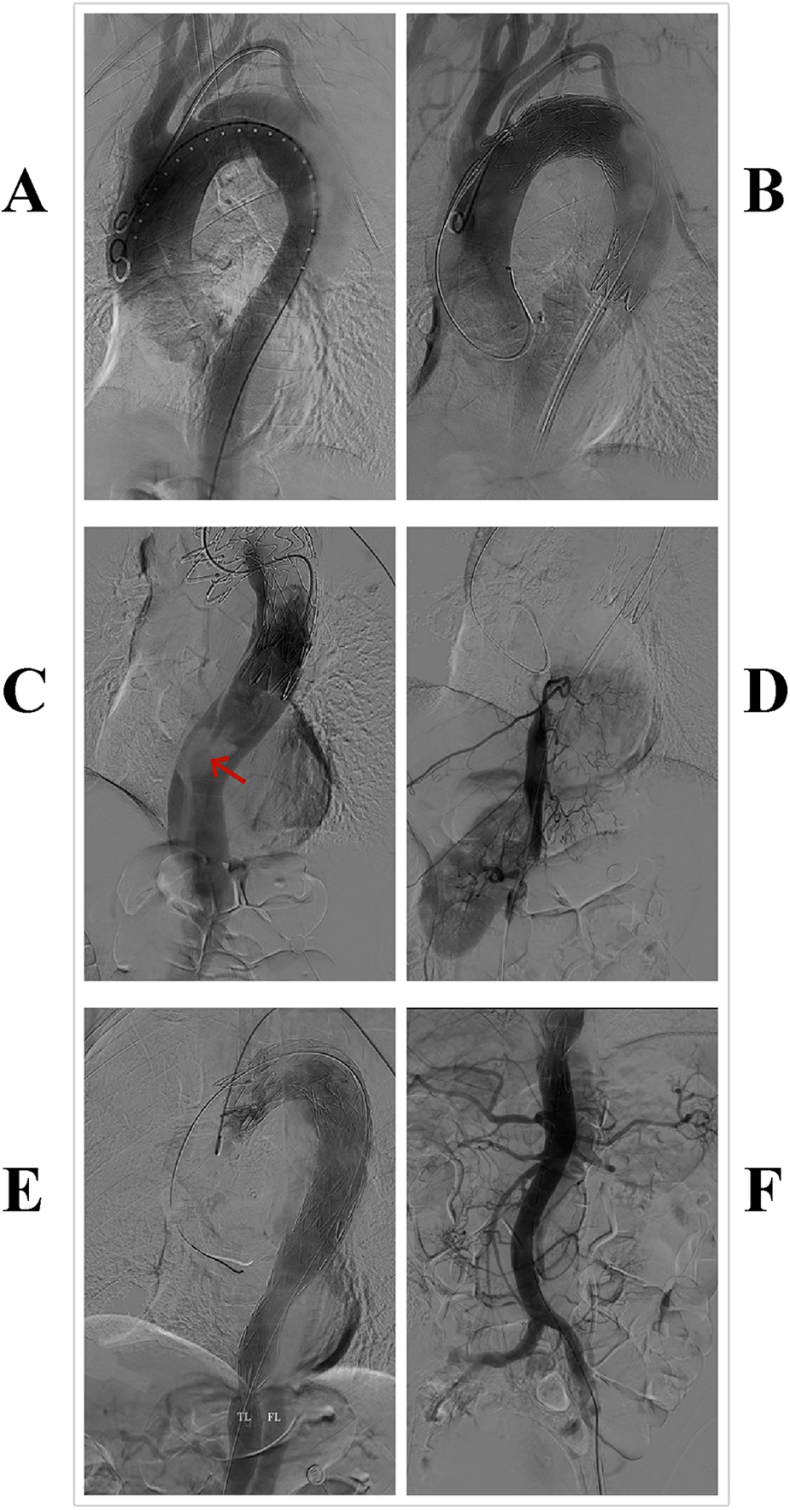
Surgical procedure: **(A)** aortic angiography. **(B,C)** After the implantation of the first endograft, angiography revealed slow blood flow in the aorta, with no visualization of abdominal organs; a tear was visible at the distal end of the graft (highlighted by a red arrow). **(D)** Guidewire insertion through the right femoral artery into the true lumen of the aorta, with subsequent angiography showing visualization of the intercostal arteries and the right renal artery. **(E,F)** After implantation of the second covered stent graft, angiography showed restored aortic blood flow velocity and visualization of visceral arteries.

During the operation, the patient was placed in the supine position, and general anesthesia was administered. Access was obtained through the left femoral artery, and angiography confirmed the correct positioning of the guidewire in the “true lumen” of the aortic, with the intimal tear located 8 mm below the origin of the left subclavian artery (Figure 2A). A 36–28 200 mm Ankura II aortic stent-graft (Lifetech Scientific Co, Ltd, Shenzhen, China) was then deployed. However, subsequent angiography showed satisfactory blood flow in the arch arteries but a decrease in flow beyond the stent, with no visibility of the visceral arteries (Figure 2B). An intimal tear was observed at the T12 vertebra level (Figure 2C), suggesting possible misplacement of the stent graft into the false lumen.

**Figure 3:**
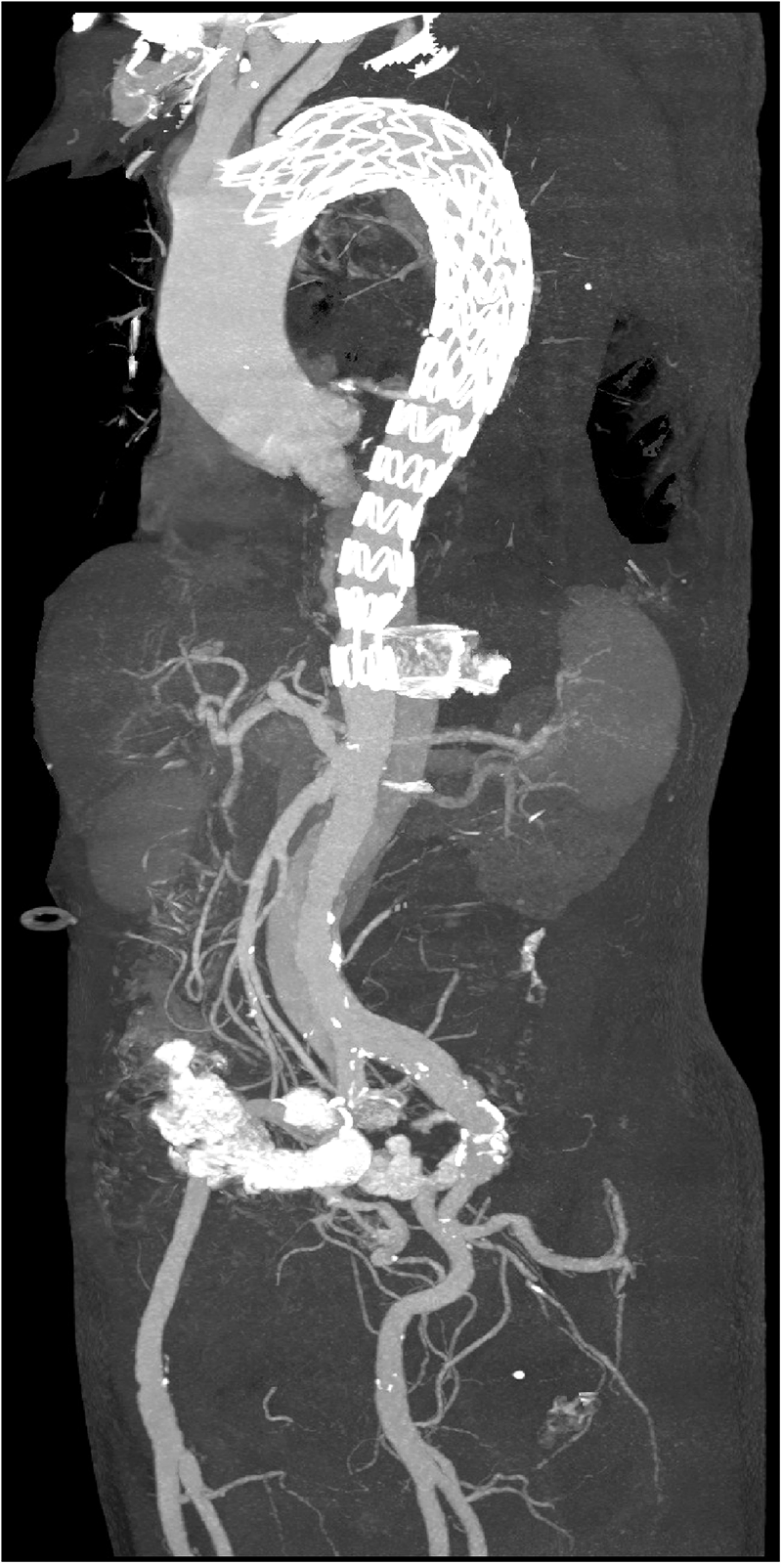
Postoperative three-dimensional reconstruction of the aorta. The distal end of the stent graft is positioned at the level of the T12 vertebra.

Due to the risk of false lumen rupture, an additional endograft was required to redirect blood flow to the true lumen. The right femoral artery was punctured, and a guidewire and a 6F guiding catheter were introduced. Angiography confirmed the position of the true lumen and visualized the left renal artery (Figure 2D). After multiple attempts, the guidewire was successfully navigated through the stent graft into the ascending aorta. A second 30–26 200 mm Ankura II aortic stent-graft was then implanted, with its distal end placed at the T12 vertebra level (Figure 2E). Follow-up angiography demonstrated restored flow in the true lumen, with the celiac trunk, superior mesenteric artery, and both renal arteries clearly visualized (Figure 2F). Postoperatively, the patient recovered successfully, with no reports of chest pain, renal failure, or paralysis, and was discharged in good health. A CTA at the one-month postoperative follow-up showed no endoleak or false lumen expansion, with satisfactory perfusion of the abdominal organs (Figure 3). Written informed consent was obtained for the publication of patient information and images.

## 3 Discussion

Endovascular repair of acute TBAD is a well-established technique known for its minimally invasive nature and its effectiveness in promoting aortic remodeling and reducing mortality rates. However, TEVAR is not without risks, and potential complications include endoleak, stent migration, endograft infection, and spinal cord ischemia (2–5). Among these, one particularly rare but severe complication is the misdeployment of the stent-graft into the false lumen. This can increase false lumen pressure, extend the intimal tear, and potentially lead to aortic rupture or inadequate perfusion of visceral organs. Prompt intervention is crucial to mitigate these life-threatening risks.

Misdeployment of the endograft into the false lumen is infrequently reported in the literature. Our review identified only six relevant case reports (Table 1), (6–11). Of these, four patients underwent total arch repair (TAR) with a frozen elephant trunk (FET), while the remaining two underwent TEVAR. Notably, FET grafts appear to have a higher likelihood of unintended deployment into the false lumen compared to TEVAR. This may be attributed to the deployment of FET grafts under direct vision, without the assistance of aortography or intravascular ultrasound.

**Table 1:** Recent cases of endograft deployment into the false lumen in aortic surgery.

No.	Authors (year)	Sex	Age	Classification of AD	Initial surgical approach	Remedial measures	Outcome
1	Tamai et al. (2019) (6)	M	40	Type A	Total arch replacement (TAR)+ FET	Additional FET	Alive
2	Takagi et al. (2021) (7)	M	83	Type A	TAR + FET	Additional stent graft	Alive
3	Plotkin et al. (2019) (8)	M	64	Type A + Aortic aneurysm	TAR + FET	Additional stent graft	Alive
4	Fujii et al. (2018) (9)	M	45	Type B	Femorofemoral bypass + TAR + FET	Enterectomy + right external iliac to superior mesenteric artery bypass + Abdominal aorta fenestration	Dead
5	Follis et Al. (2009) (10)	M	50	Type B	TEVAR	Removal of the endograft from the FL	Alive
6	Ma et al. (2016) (11)	M	42	Type B	TEVAR	Additional stent graft + coils embolization of the FL	Alive

AD, aortic dissection; TAR, total arch repair; FET, frozen elephant trunk; FL, false lumen.

In reports by Tamai et al. (6), Daichi et al. (7), and Plotkin et al. (8), intraoperative transesophageal echocardiography successfully detected misdeployment of the FET graft, leading to immediate corrective action. Another FET graft was deployed at the distal end of the misdeployed graft to redirect blood flow into the true lumen. Thanks to these timely interventions, all patients recovered and were discharged without severe complications. However, in the case reported by Masahiko et al., the misdeployment went undetected intraoperatively and was only discovered two days later when the patient developed lactic acidosis. This necessitated extensive surgical intervention, including enterectomy, extra-anatomical bypass from the right external iliac artery to the superior mesenteric artery, and abdominal aorta fenestration. Unfortunately, the patient ultimately succumbed to multiorgan failure due to severe intestinal ischemia.

The case reported by Ma et al. (11) shares similarities with our own. Ma et al. successfully utilized the Outback LTD Re-Entry Catheter to puncture the septum below the previously misdeployed endograft, thereby establishing a pathway through the true lumen of the descending aorta. Following this, another endograft was deployed, redirecting blood flow back into the true lumen and leading to the patient's recovery and discharge. In our case, we established a guidewire pathway through the intimal tear at the distal end of the misdeployed endograft and subsequently deployed a covered stent at this site, successfully redirecting blood flow back into the true lumen. The patient recovered without any complications and was discharged in good health.

Based on our literature review (1, 12), we hypothesize that the distal implantation of the endograft into the false lumen, in this case, may have occurred due to the guidewire entering the false lumen through a distal intimal tear in the descending aorta or iliac artery, subsequently re-entering the true lumen via the proximal primary intimal tear. Given that pressure within the true lumen is higher than in the false lumen and considering the narrowness of the true lumen in the descending aorta, the guidewire is more likely to pass into the false lumen through the distal tear. Since the initial part of the false lumen is often a blind end, the guidewire can only reach the ascending aorta by re-entering the true lumen through the proximal intimal tear.

The detection of the stent graft's misdeployment into the false lumen during the procedure highlights the importance of post-stent deployment angiographic evaluation to ensure adequate visceral organ perfusion. Accurately identifying the aortic true and false lumens is crucial to prevent unintentional guidewire entry into the false lumen. To mitigate this risk, several strategies are recommended, including continuous low-dose angiography to distinguish between the true and false lumens (10), bilateral femoral artery punctures with sequential placement of angiographic catheters in both lumens (13), and the use of intravascular ultrasound to confirm the positions of the true and false lumens, especially in challenging scenarios (14). These measures collectively enhance procedural safety and contribute to better outcomes in complex aortic interventions.

## 4 Conclusion

Accidental stent implantation into the false lumen during TEVAR is a rare but serious complication. Intraoperative angiography plays a crucial role in rapidly and accurately identifying this complication by detecting insufficient perfusion of abdominal organs. The use of intravascular ultrasound can help reduce the incidence of such complications. Endovascular repair remains the preferred method for emergency management, as it can swiftly and effectively redirect blood flow back to the true lumen.

## Data Availability

The raw data supporting the conclusions of this article will be made available by the authors, without undue reservation.
